# Inhibition of miR-19a partially reversed the resistance of colorectal cancer to oxaliplatin via PTEN/PI3K/AKT pathway

**DOI:** 10.18632/aging.102929

**Published:** 2020-03-25

**Authors:** Ye Zhang, Xinxin Liu, Junying Zhang, Yuanyuan Xu, Jie Shao, Yue Hu, Peng Shu, Haibo Cheng

**Affiliations:** 1 Department of Oncology, Jiangsu Province Hospital of Chinese Medicine, Affiliated Hospital of Nanjing University of Chinese Medicine, Nanjing 210029, Jiangsu Province, China; 2 Department of General Surgery, Jiangsu Province Hospital of Chinese Medicine, Affiliated Hospital of Nanjing University of Chinese Medicine, Nanjing 210029, Jiangsu Province, China; 3 Clinical Cancer Research Center, Jiangsu Cancer Hospital and Jiangsu Institute of Cancer Research and The Affiliated Cancer Hospital of Nanjing Medical University, Nanjing 210009, Jiangsu Province, China; 4 The First Clinical Medical College, Nanjing University of Chinese Medicine, Nanjing 210023, Jiangsu Province, China

**Keywords:** anti-miR-19a, PTEN, PI3K, AKT, oxaliplatin

## Abstract

Oxaliplatin is a platinum-based chemotherapeutic drug that is effective and commonly used in the treatment of colorectal cancer (CRC). However, long-term use of oxaliplatin usually induces significant drug resistance. It is urgent to develop strategies to reverse the oxaliplatin resistance to CRC cells. In the present study, we established the model of oxaliplatin-resistant CRC cell lines (SW480/R and HT29/R) through continuous treatment of SW480 and HT29 cells with oxaliplatin. Results of qRT-PCR analysis showed that expression of miR-19a was significantly increased in SW480/R and HT29/R compared to their parental SW480 and HT29. However, combination treatment with anti-miR-19a, an antisense oligonucleotide of miR-19a, was found to resensitize SW480/R and HT29/R cells to oxaliplatin treatment. In the mechanism research, we found that anti-miR-19a increased the expression of PTEN and thus inhibited the phosphorylation of PI3K and AKT in SW480/R and HT29/R cells. As a result, mitochondrial apoptosis induced by oxaliplatin was expanded. We demonstrated that PTEN was the target of miR-19a and inhibition of miR-19a partially reversed the resistance of colorectal cancer to oxaliplatin via PTEN/PI3K/AKT pathway.

## INTRODUCTION

Colorectal cancer (CRC) is the third most common type of malignant tumor with poor prognosis in the world [1]. Nowadays, it is still difficult to make an early diagnosis for CRC. Therefore, most patients were diagnosed as advanced CRC at their first interview with a doctor. Furthermore, because of the high invasion and early metastasis of CRC, the success rate of surgery is not satisfactory [2]. For the advanced CRC patients whose tumors are unresectable, systematic chemotherapy is irreplaceable and valuable [3–5]. However, CRC cells usually acquire drug resistance through the repeated use of chemotherapeutic drugs [6–8]. It is urgent to explore the potential mechanisms of the development of drug resistance.

Oxaliplatin, a third-generation of platinum compound, is known to induce cross-linking of cancer cell DNAs and thus causes their apoptotic cell death [9–12]. Although oxaliplatin is the first-line platinum-based compound to show efficacy in the treatment of CRC, virtually all metastatic CRC cells eventually become resistant to oxaliplatin [13–16]. Thus, intervention that attenuates the resistance of oxaliplatin is required in the treatment of CRC.

MicroRNAs (miRNAs) are small and endogenous non-coding RNAs that negatively regulate the expression of downstream targeted mRNAs through binding to the 3′ untranslated region (3′ UTR) of the mRNAs [17–19]. Previous studies have proved that dysregulation of miRNA expression leads to the proliferation, metastasis and drug resistance of cancer cells [20–26]. Among these cancer-related miRNAs, miR-19a is reported to act as an important factor that is responsible for drug resistance in several cancers. Thus, miR-19a may be a promising target for improving the chemotherapy [27, 28]. In the present study, we established the oxaliplatin resistance models on SW480 and HT29 which are the CRC cell lines. We aimed to explore the potential association between oxaliplatin resistance and the specific miRNA of miR-19a in CRC.

## RESULTS

### Establishment of oxaliplatin-resistant CRC cells

**Figure 1:**
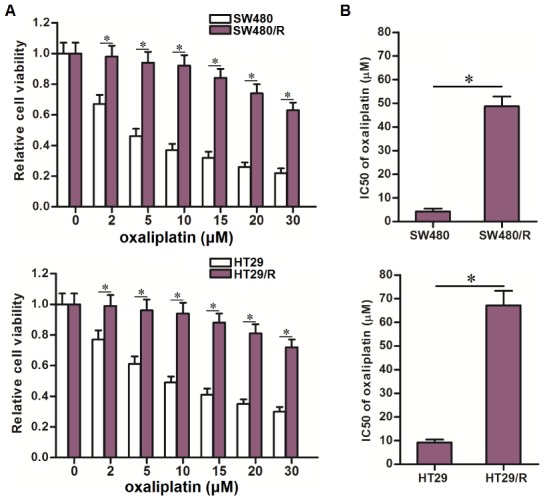
**Oxaliplatin resistance of SW480/R and HT29/R.** (**A**) MTT assays were performed to evaluate the cytotoxicity of different concentrations of oxaliplatin (0~30 μM) to SW480, SW480/R, HT29 and HT29/R. *P<0.05. (**B**) IC50 of oxaliplatin to SW480, SW480/R, HT29 and HT29/R. *P<0.05.

To study the resistance of oxaliplatin on CRC, we continuously exposed the CRC cell lines SW480 and HT29 to oxaliplatin. The obtained cells were named as SW480/R and HT29/R. We then treated these SW480/R and HT29/R cells and their parental SW480 and HT29 cells with different concentrations of oxaliplatin to compare their drug sensitivity. Compared to the SW480 and HT29 cells, the SW480/R and HT29/R cells exhibited significant lower sensitivity to various concentrations of oxaliplatin (Figure 1A). We then calculated that IC50 of oxaliplatin to SW480/R was 10.3 fold higher than that to SW480 cells. Meanwhile, IC50 of oxaliplatin to HT29/R was 7.2 fold higher than that to HT29 cells (Figure 1B). We confirmed that our established SW480/R and HT29/R were resistant to oxaliplatin.

### Overexpression of miR-19a is responsible for the oxaliplatin resistance of SW480/R and HT29/R

**Figure 2:**
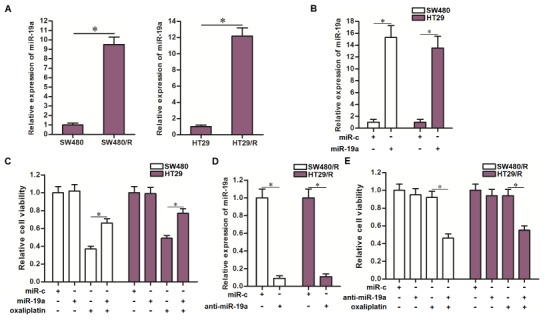
**Effect of miR-19a on regulating the oxaliplatin sensitivity of CRC cells.** (**A**) QRT-PCR analysis was performed to detect the expression of miR-19a in SW480, SW480/R, HT29 and HT29/R. *P<0.05. (**B**) Transfection with miR-19a mimics (50 pmol/ml) increased the cellular level of miR-19a in SW480 and HT29 cells. *P<0.05. (**C**) Transfection with miR-19a mimics (50 pmol/ml) decreased the sensitivity of SW480 and HT29 cells to oxaliplatin (10 μM) treatment. *P<0.05. (**D**) Transfection with anti-miR-19a (50 pmol/ml) decreased the cellular level of miR-19a in SW480/R and HT29/R cells. *P<0.05. (**E**) Transfection with anti-miR-19a (50 pmol/ml) increased the sensitivity of SW480/R and HT29/R cells to oxaliplatin (10 μM) treatment. *P<0.05.

Results of qRT-PCR analysis showed that SW480/R and HT29/R cells expressed significantly higher level of miR-19a compared to their parental SW480 and HT29 cells (Figure 2A). To investigate the association between miR-19a and oxaliplatin resistance of CRC, we overexpressed the miR-19a in routine SW480 and HT29 cells through transfection with miR-19a mimics (Figure 2B). We then found that overexpression of miR-19a decreased the sensitivity of routine SW480 and HT29 cells to oxaliplatin treatment (Figure 2C). On the other hand, we inhibited the function of miR-19a in SW480/R and HT29/R cells through transfection with miR-19a antisense oligonucleotide (Figure 2D). We then found that knockdown of miR-19a obviously increased the sensitivity of SW480/R and HT29/R cells to oxaliplatin treatment (Figure 2E). Taken together, these data indicated overexpression of miR-19a is responsible for the induction of oxaliplatin resistance of CRC. Furthermore, inhibition of miR-19a function was conducive to reverse the oxaliplatin resistance of CRC.

### Anti-miR-19a increases the expression of PTEN in SW480/R and HT29/R cells

**Figure 3:**
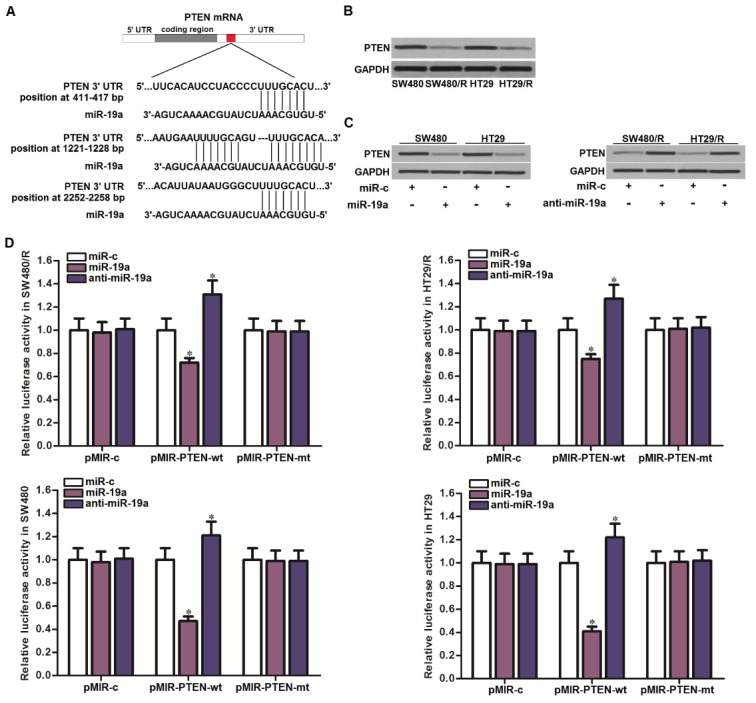
**MiR-19a targeted PTEN in CRC.** (**A**) Multiple regions of PTEN mRNA 3’ UTR exist potential binding sites paired with miR-19a. (**B**) Western blot assays were performed to test the expression of PTEN in SW480, SW480/R, HT29 and HT29/R cells. (**C**) Western blot assays were performed to test the effect of miR-19a (50 pmol/ml) and anti-miR-19a (50 pmol/ml) on changing the expression of PTEN in SW480, SW480/R, HT29 and HT29/R cells. (**D**) Luciferase reporter assays were performed to test the effect of miR-19a (50 pmol/ml) and anti-miR-19a (50 pmol/ml) on changing the luciferase activities of the pMIR reporters containing PTEN 3’-UTR in SW480, SW480/R, HT29 and HT29/R cells. *P<0.05 *vs.* miR-c group.

To explore the mechanism by which miR-19a partially determined the oxaliplatin resistance of CRC, we searched the potential target of miR-19a through the public miRNA prediction databases of TargetScan, miRanda, and PicTar. Among the candidate targeted genes, PTEN was commonly predicted by all of these databases. The potential complementary site paired with miR-19a and PTEN was shown in Figure 3A. On the other hand, we detected obviously lower level of PTEN in SW480/R and HT29/R cells compared to the routine SW480 and HT29 cells (Figure 3B). Together the results of Figure 2A, we predicted that PTEN was the target of miR-19a. To test this prediction, we changed the level of miR-19a before evaluation of PTEN expression in CRC cell lines. In SW480 and HT29 cells, overexpression of miR-19a was found to decrease the expression of PTEN, and in SW480/R and HT29/R cells, knockdown of miR-19a was found to increase the expression of PTEN (Figure 3C). On the other hand, results of luciferase reporter assays showed that transfection with miR-19a significantly inhibited the luciferase activities of the reporters contained wild type PTEN 3′-UTR, whereas transfection with anti-miR-19a increased the luciferase activities of the reporters (Figure 3D). Taken together, we demonstrated that PTEN was the target of miR-19a. SW480/R and HT29/R cells overexpressed the miR-19a to reduce the expression of PTEN.

### Expression level of PTEN partially determines the oxaliplatin sensitivity of CRC

**Figure 4:**
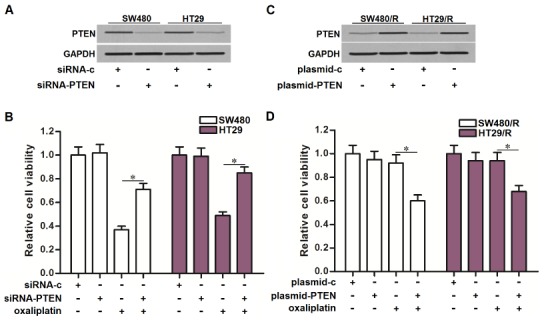
**Effect of PTEN on regulating the oxaliplatin sensitivity of CRC cells.** (**A**) Transfection with siRNA-PTEN (50 pmol/ml) decreased the expression of PTEN in SW480 and HT29 cells. (**B**) Transfection with siRNA-PTEN (50 pmol/ml) decreased the sensitivity of SW480 and HT29 cells to oxaliplatin (10 μM) treatment. *P<0.05. (**C**) Transfection with plasmid-PTEN (2 μg/ml) increased the expression of PTEN in SW480/R and HT29/R cells. (**D**) Transfection with plasmid-PTEN (2 μg/ml) increased the sensitivity of SW480/R and HT29/R cells to oxaliplatin (10 μM) treatment. *P<0.05.

To explore the role of PTEN in determining the oxaliplatin sensitivity of CRC, we directly knockdown the PTEN in SW480 and HT29 cells by using its specific siRNA (siRNA-PTEN) (Figure 4A). We then found that treatment with siRNA-PTEN significantly decreased the sensitivity of SW480 and HT29 cells to oxaliplatin (Figure 4B). On the other hand, we overexpressed the expression of PTEN in SW480/R and HT29/R cells by using the eukaryotic expression vector (plasmid-PTEN) (Figure 4C). Interestingly, treatment with plasmid-PTEN obviously reversed the oxaliplatin resistance of SW480/R and HT29/R cells (Figure 4D). We demonstrated that expression level of PTEN partially determined the oxaliplatin sensitivity of CRC. Increase of PTEN expression was conducive to reverse the oxaliplatin resistance of CRC.

### Anti-miR-19a partially reverses the oxaliplatin resistance of CRC through upregulation of PTEN

**Figure 5:**
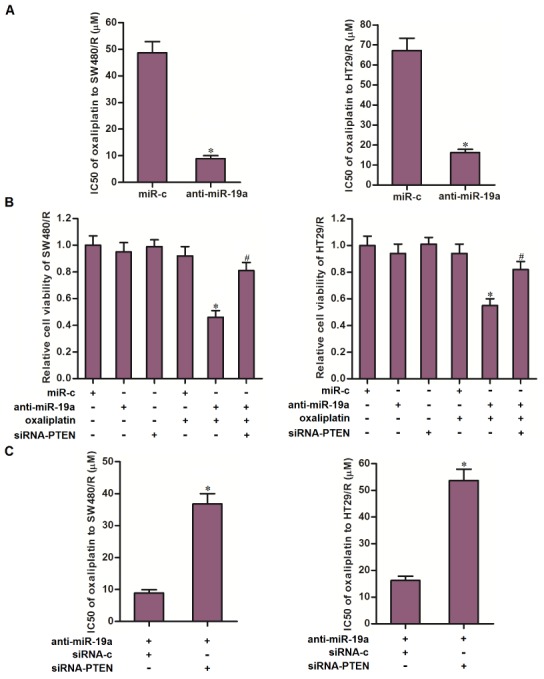
**Anti-miR-19a partially reversed the oxaliplatin resistance of CRC cells through the PTEN pathway.** (**A**) Combination treatment with anti-miR-19a (50 pmol/ml) decreased the IC50 of SW480/R and HT29/R to oxaliplatin. *P<0.05 *vs.* miR-c group. (**B**) Transfection with siRNA-PTEN (50 pmol/ml) increased the cell viability of SW480/R and HT29/R cells which were co-treated with anti-miR-19a (50 pmol/ml) and oxaliplatin (10 μM). *P<0.05 *vs.* oxaliplatin+miR-c group. ^#^*P*<0.05 *vs.* oxaliplatin+anti-miR-19a group. (**C**) Transfection with siRNA-PTEN (50 pmol/ml) abolished the effect of anti-miR-19a on reducing the IC50 of oxaliplatin to SW480/R and HT29/R. *P<0.05 *vs.* anti-miR-19a+siRNA-c group.

Results of MTT assays showed that transfection with anti-miR-19a reduced the IC50 of oxaliplatin to SW480/R by 81.7 percent and HT29/R by 75.9 percent (Figure 5A). We thus demonstrated that co-treatment with anti-miR-19a can partially reverse the oxaliplatin resistance of CRC. To investigate whether the mechanism by which anti-miR-19a resensitized SW480/R and HT29/R cells to oxaliplatin was dependent on the upregulation of PTEN, we knocked down the PTEN in SW480/R and HT29/R cells by using siRNA-PTEN when they were co-treated with oxaliplatin and anti-miR-19a. Results of MTT assays showed that knockdown of PTEN abolished the effect of anti-miR-19a on sensitizing the SW480/R and HT29/R cells to oxaliplatin treatment (Figure 5B). Specifically, siRNA-PTEN significantly increased the IC50 of oxaliplatin to anti-miR-19a-treated SW480/R and HT29/R cells (Figure 5C). Taken together, these results indicated that co-treatment with anti-miR-19a can partially reverse the oxaliplatin resistance of CRC through upregulation of PTEN.

### Anti-miR-19a targets the PTEN/PI3K/AKT pathway to resensitize the oxaliplatin-induced apoptosis in SW480/R and HT29/R

**Figure 6:**
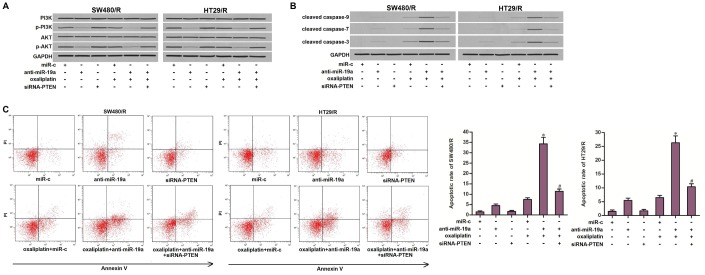
**Anti-miR-19a enhanced the oxaliplatin-induced apoptosis through the PTEN/PI3K/AKT pathway.** (**A**) Western blot assays were performed to evaluate the effect of anti-miR-19a (50 pmol/ml) and siRNA-PTEN (50 pmol/ml) on affecting the phosphorylation of PI3K and AKT in SW480/R and HT29/R cells. (**B**) Western blot assays were performed to evaluate the effect of anti-miR-19a (50 pmol/ml) and siRNA-PTEN (50 pmol/ml) on affecting the activation of caspase-9, -7 and -3 which was dependent by oxaliplatin in SW480/R and HT29/R cells. (**C**) Anti-miR-19a (50 pmol/ml) increased the apoptotic rate of SW480/R and HT29/R cells which were treated with oxaliplatin (10 μM). *P<0.05 *vs.* oxaliplatin+miR-c group. ^#^*P*<0.05 *vs.* oxaliplatin+anti-miR-19a group.

Since the PTEN is a natural inhibitor of PI3K and AKT [29], we next tested the association between the anti-miR-19a and the PTEN/PI3K/AKT signaling pathway. As shown in Figure 6A, single treatment with oxaliplatin can not alter the activity of PI3K or AKT. However, treatment with anti-miR-19a inhibited the phosphorylation of PI3K and AKT. Furthermore, we observed that transfection with anti-miR-19a significantly promoted the activation of caspase-9, -7, -3 in the oxaliplatin-treated SW480/R and HT29/R cells (Figure 6B). As a result, we showed that anti-miR-19a significantly enhanced the oxaliplatin-induced apoptosis in SW480/R and HT29/R cells (Figure 6C). These results demonstrated that anti-miR-19a can sensitize the oxaliplatin-induced apoptosis through the PTEN/PI3K/AKT pathway in the oxaliplatin-resistant CRC cells.

## DISCUSSION

Oxaliplatin is a third-generation of platinum-based antineoplastic drug that is commonly used for the treatment of gastrointestinal cancers including CRC. However, development of acquired drug resistance of CRC usually leads to the failure of oxaliplatin treatment [30–32]. Therefore, it is urgent to explore the potential mechanism of the formation of drug resistance. In the present study, we continuously exposed the CRC cell lines to oxaliplatin. We then found that long-term exposure to oxaliplatin can induce significant drug resistance in CRC.

Studies have reported that dysregulation of miRNA expression leads to the drug resistance and poor prognosis in various digestive tract cancers including CRC, oral cancer and gastric cancer [33–36]. Among these cancer-related miRNAs, miR-19a was found to be significantly upregulated in our established oxaliplatin-resistant CRC cell lines. Previous studies have reported that miR-19a acts as a tumor promoter in multiple cancers. For instance, miR-19a was overexpressed in bladder cancer cells. It promoted invasion and epithelial to mesenchymal transition of bladder cancer cells by targeting RhoB [37]. Furthermore, overexpression of miR-19a was an underlying risk of poor prognosis in many human malignancies, especially in osteosarcoma. Elevated miR-19a expression was linked to the potential of lymph node metastasis [38]. More importantly, studies indicated that overexpression of miR-19a contributed to chemoresistance to multiple cancers including ovarian cancer and non-small cell lung cancer [39, 40]. Therefore, miR-19a was a potential target that may be responsible for the drug resistance of CRC.

As miR-19a has been proven to a potent tumor promoter, we next explored the association between miR-19a overexpression and resistance of oxaliplatin to CRC. For this purpose, we knocked down the miR-19a in oxaliplatin-resistant CRC cells. We then found that knockdown of miR-19a partially reversed the oxaliplatin resistance of CRC. On the other hand, increasing the expression of miR-19a in routine CRC cells induced the oxaliplatin resistance of CRC. These results indicated that miR-19a expression level was closely associated with oxaliplatin sensitivity to CRC. Furthermore, anti-miR-19a can be used as a sensitizer to reverse the resistance of oxaliplatin.

Phosphatase and tensin homologue (PTEN) has been fully proved to be a potent tumor suppressor in multiple cancers. However, PTEN is downregulated in many cancers. Moreover, change of PTEN expression is one of the critical factors for cancer development and drug resistance [41–43]. In the PTEN signaling pathway, PTEN can inhibit the phosphorylation of PI3K and the subsequent generation of phosphatidylinositol-3,4,5-trisphosphate (PIP3), which in turn triggers the AKT. As activation of AKT inhibits apoptosis, cellular PTEN can promote the cell death of cancer cells through the PI3K/AKT pathway [44–46].

In this study, we found that PTEN was downregulated in oxaliplatin-resistant CRC cells. Absence of PTEN increased the phosphorylation of PI3K and AKT. Therefore, oxaliplatin-resistant CRC cells exhibited lower response to oxaliplatin-induced cell death compared to the routine CRC cells. Furthermore, we found that the mechanism by which PTEN was downregulated in oxaliplatin-resistant CRC cells was dependent on the overexpression of miR-19a. That is to say, PTEN was the target of miR-19a in CRC. Our results indicated that treatment with anti-miR-19a can increase the PTEN expression and thus inhibit the phosphorylation of PI3K and AKT in the oxaliplatin-resistant CRC cells. Finally, treatment with anti-miR-19a resensitized the oxaliplatin-resistant CRC cells to oxaliplatin-induced apoptosis.

## CONCLUSIONS

This study indicated the effect of anti-miR-19a on reversing the oxaliplatin resistance of CRC. Inhibition of PI3K/AKT pathway through the miR-19a/PTEN axis may represent a potential strategy for attenuating the oxaliplatin resistance of CRC.

## MATERIALS AND METHODS

### Cell culture

The human CRC cell lines SW480 and HT29 were purchased from the Institute of Biochemistry and Cell Biology, Chinese Academy of Sciences (Shanghai, China). Cells were cultured in RPMI-1640 medium (Gibco, Carlsbad, CA, USA) supplemented with 10% fetal bovine serum (FBS). Oxaliplatin-resistant SW480 and HT29 (SW480/R and HT29/R) cells were obtained by continuous exposure of HT29 and SW480 cells to increasing concentrations of oxaliplatin from 0.2 μM to 2 μM for 6 months. All of the cells were cultured at 37°C in a humidified incubator with 5% CO_2_.

### Transient transfection

Human miR-19a mimics, miR-19a antisense oligonucleotide (anti-miR-19a), control oligonucleotides (miR-c), siRNA targeted PTEN (siRNA-PTEN), control siRNA (siRNA-c) were purchased from Genechem Co., Ltd. (Shanghai, China). PTEN eukaryotic expression plasmid was generated by cloning the open reading frame of the PTEN gene into the pcDNA3.1 plasmid (Life Technologies, Carlsbad, CA, USA). For transfection, cells were seeded into six-well plates, with the density of 4×10^5^ cells/well. Subsequently, the above RNA oligonucleotides or plasmid was transfected into cells with Lipofectamine^®^2000 (Thermo Fisher Scientific, Inc., Carlsbad, CA, USA) according to the manufacturer’s instruction.

### Quantitative real-time polymerase chain reaction (qRT-PCR)

Cellular total RNAs were extracted by using Trizol reagent (Invitrogen, Carlsbad, CA, USA). cDNA was reversely transcribed by using the extracted RNAs and One Step PrimeScript miRNA cDNA Synthesis Kit (TaKaRa, Dalian, China). Expression of miR-19a was detected through qRT-PCR assay by using SYBR Green PCR kit (Toyobo, Japan). QPCR primer of miR-19a is as follows: 5′-TGTGCAAATCTATGCAAAACTGA-3′. U6 small nuclear RNA (snRNA U6) was used as the internal reference to determine the relative expression of miR-19a through the 2^−ΔΔCT^ method.

### Cell viability assay

Cells were transfected and seeded into 96-well plates at a density of 5×10^3^ cells/well with 100 μl culture medium. After overnight incubation, cells were then treated with different concentrations of oxaliplatin (0~30 μmol/L) (Sigma-Aldrich, St. Louis, MO, USA) for 48 h. Subsequently, 20 μl MTT reagent (5 mg/mL; Sigma-Aldrich) was added to the culture medium and the cells were incubated for another 4 h. The cells were then suspended in 150 μl dimethyl sulfoxide followed by detection of the absorbance at 570 nm on a microplate reader (Bio-Tek Instruments, Inc., Norcross, GA, USA). Half maximal inhibitory concentration (IC50) of oxaliplatin was calculated according to the cell viability curve.

### Luciferase reporter assay

PTEN 3′ UTR fragment which was predicted as miR-19a binding site was amplified from cDNA of CRC cells and inserted into pMIR Firefly luciferase reporters (Ambion, Carlsbad, CA, USA). pMIR reporter with mutant PTEN 3′ UTR was conducted by using QuikChange Site-Directed Mutagenesis kit (Stratagene, Missouri, TEX, USA). For luciferase reporter assay, cells were co-transfected with pMIR reporter and miR-c, miR-19a or anti-miR-19a. 24 h after transfection, cells were collected and lysed. Luciferase activities were then confirmed by using dual-luciferase reporter assay system (Promega, Madison, WI, USA) according to the manufacturer’s instruction.

### Western blot analysis

Cellular total proteins were extracted by using RIPA lysis buffer (Cell Signaling Technology Inc., Danvers, MA, USA). 50 μg of the extracted proteins were separated by 10 % sodium dodecyl sulfate polyacrylamide gel electrophoresis (SDS-PAGE) and transferred to a PVDF membrane (Millipore, Billerica, MA, USA). Membranes were then probed with primary antibodies (anti-PTEN (dilution: 1:1000, catalog number: #9188), anti-PI3K (dilution: 1:1000, catalog number: #4249), anti-AKT (dilution: 1:1000, catalog number: #4691), anti-p-PI3K(Tyr458) (dilution: 1:1000, catalog number: #17366), anti-p-AKT(Ser473) (dilution: 1:1000, catalog number: #4060), anti-cleaved caspase-9 (dilution: 1:1000, catalog number: #20750), anti-cleaved caspase-7 (dilution: 1:1000, catalog number: #8438), anti-cleaved caspase-3 (dilution: 1:1000, catalog number: #9664) and anti-GAPDH (dilution: 1:1000, catalog number: #5174)) (Cell Signaling Technology Inc) overnight. Subsequently, the membranes were incubated with a horseradish peroxidase-conjugated secondary antibody for 2 h at room temperature. Blots were visualized by using enhanced chemiluminescence detection kit (Pierce, Rockford, IL, USA).

### Cell apoptosis detection

Cells were collected and washed with PBS. Subsequently, cells were stained with Annexin V-FITC and propidium iodide (BD Pharmingen, San Diego, CA, USA) away from light. Samples were then analyzed by flow cytometry. Annexin V-positive cell population was calculated as the apoptotic cells.

### Statistical analysis

All data are obtained from at least three independent experiments and represented as the mean ± standard deviation (SD). Non-paired t test was used to estimate the statistical differences between two groups. One-way analysis of varianvce (ANOVA) was applied to verify differences among three or more groups. Statistical analysis was performed by using SPSS 16.0 software (SPSS Inc., Chicago, IL, USA). *P* < 0.05 was considered to be statistically significant.
